# Association between antipsychotic/antidepressant drug treatments and hospital admissions in schizophrenia assessed using a mental health case register

**DOI:** 10.1038/npjschz.2015.35

**Published:** 2015-10-21

**Authors:** Rudolf N Cardinal, George Savulich, Louisa M Mann, Emilio Fernández-Egea

**Affiliations:** 1 Behavioural and Clinical Neuroscience Institute, Department of Psychiatry, University of Cambridge, Cambridge, UK; 2 Cambridgeshire and Peterborough NHS Foundation Trust, Elizabeth House, Fulbourn Hospital, Cambridge, UK

## Abstract

**Background:**

The impact of psychotropic drug choice upon admissions for schizophrenia is not well understood.

**Aims:**

To examine the association between antipsychotic/antidepressant use and time in hospital for patients with schizophrenia.

**Methods:**

We conducted an observational study, using 8 years’ admission records and electronically generated drug histories from an institution providing secondary mental health care in Cambridgeshire, UK, covering the period 2005–2012 inclusive. Patients with a coded ICD-10 diagnosis of schizophrenia were selected. The primary outcome measure was the time spent as an inpatient in a psychiatric unit. Antipsychotic and antidepressant drugs used by at least 5% of patients overall were examined for associations with admissions. Periods before and after drug commencement were compared for patients having pre-drug admissions, in mirror-image analyses correcting for overall admission rates. Drug use in one 6-month calendar period was used to predict admissions in the next period, across all patients, in a regression analysis accounting for the effects of all other drugs studied and for time.

**Results:**

In mirror-image analyses, sulpiride, aripiprazole, clozapine, and olanzapine were associated with fewer subsequent admission days. In regression analyses, sulpiride, mirtazapine, venlafaxine, and clozapine–aripiprazole and clozapine–amisulpride combinations were associated with fewer subsequent admission days.

**Conclusions:**

Use of these drugs was associated with fewer days in hospital. Causation is not implied and these findings require confirmation by randomized controlled trials.

## Introduction

Admissions to psychiatric inpatient units are a potential marker of disease severity in schizophrenia, and a burden on the individual. UK guidelines emphasize avoiding admission, and antipsychotic drugs as the mainstay of treatment.^1^ Randomized^2,3^ and non-randomized^4–8^ studies have examined the effect of some antipsychotics on admissions. However, information on admission reduction is sparser across the full range of drugs used clinically, and in relation to multiple-drug strategies such as clozapine augmentation^9,10^ and antidepressant addition.^11^

We sought to examine the impact of individual antipsychotic drugs but also of commonly prescribed antidepressants and clozapine augmentation strategies on hospital admissions in patients with schizophrenia, using a naturalistic clinical sample across an 8-year period.

We examined the anonymized electronic records of patients with schizophrenia within an NHS Trust. We investigated the associations between the antipsychotics/antidepressants used and time spent in hospital. To examine the impact of drug initiation on patients with relatively severe schizophrenia, we conducted mirror-image analyses examining the duration of admissions before and after drug initiation, in all patients who had been admitted during the pre-drug period, controlling for overall changes in admission rate. To account for the use of more than one drug and to address additional confounds, we also conducted regression analyses to model admission durations by drug prescription, across all patients and drugs.

## Materials and methods

### Data sources

Cambridgeshire and Peterborough NHS Foundation Trust (CPFT) provides secondary mental health care to Cambridgeshire (population ~800,000).^12^ Its electronic records from 2005 to 2012 were de-identified using CRIS^13^ into a research database (UK NHS National Research Ethics Service reference 12/EE/0407). Unstructured text, such as letters and discharge summaries, were searched using GATE^14^ natural language processing (NLP) software for drug names and common misspellings in a grammatical context indicating current use, generating drug histories.^15^ The principal data sources were electronic psychiatric admission logs, autogenerated drug histories, and coded ICD-10 diagnoses.^16^ Diagnoses were assigned by clinicians (though sometimes subsequently recorded in structured fields by administrative staff) and clinicians were the predominant authors of free-text documents referencing medication use. Admission and discharge information was typically recorded by administrative staff. No electronic prescribing system was in use, necessitating the use of computer-generated drug histories.

### Inclusion and exclusion criteria and measures of illness duration

We studied patients for whom an ICD-10 diagnosis within ‘F20’ (schizophrenia) had been recorded in a structured ‘diagnosis’ field at some point. The first such entry was taken as the date of diagnosis of schizophrenia. In some cases, electronic diagnoses had been entered with retrospective dates. For patients who had died, time after death was excluded.

### Dependent variables

The primary dependent variable was the number of days spent as an inpatient (discharge date minus admission date, summed across admissions).

### Drugs analyzed

We searched for first-generation and second-generation antipsychotics (FGAs, SGAs), plus all UK-licensed antidepressants. We restricted further analyses to drugs used by ⩾5% of patients. In regression analyses, to reduce confounding from co-prescription, we also included benzodiazepines/Z-drugs and mood stabilizers used by ⩾5% of patients. We calculated a median dose (as the median of all patients’ median daily doses).

### Precision and recall

Precision and recall were measured for clozapine (see Supplementary Methods).

### Mirror-image comparisons of admission rates during pre-drug and post-drug periods

**Figure 1:**
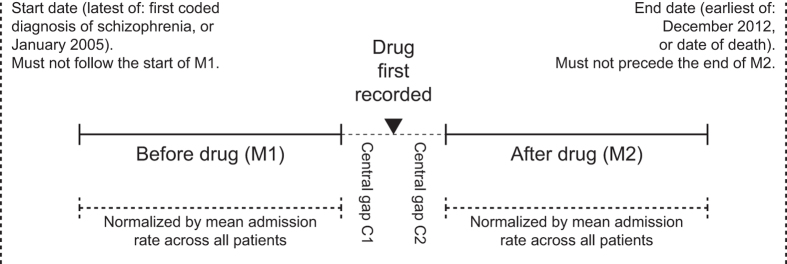
Illustration of the mirror-image design. For a given drug, the number of admission days for a given patient was calculated for a period before the first recorded use of the drug (*M*_1_, either 1 or 2 years), and a period of identical duration afterwards (*M*_2_). These rates were then corrected for the overall admission rates, for all patients with schizophrenia, during the same *M*_1_ and *M*_2_ periods (see text). Patients were excluded who had no admissions falling in the M_1_ period, thus selecting for patients with relatively severe disease. A central gap (C_1_+C_2_, each 30 days) was excluded to reduce the effects of regression to the mean and errors caused by small inaccuracies in the temporal recording of drugs.

We calculated a pre- versus post-drug comparison using a mirror-image design^17^ (Figure 1), for all drugs studied. For each patient, we calculated a start date (the study start date or their first diagnosis of schizophrenia, whichever was later), an end date (the study end date or their date of death if they had died, whichever was earlier), and the ‘first use’ date for each drug. Because of potential temporal imprecision in the exact first-use date, and to reduce the impact of regression to the mean, we excluded a central period C either side of the first-use date (giving a central gap of 2C), and then looked at a mirrored period *M* either side of that central gap (Figure 1). We examined a temporally narrow period (*M*=1 year) and a temporally broader period (*M*=2 years).

We excluded any patient if their mirror period was not within the start–end date range (i.e., if they had taken the drug soon after diagnosis, lacking sufficient pre-drug time, or if their first-use date was close to their end date, lacking sufficient post-drug time). Thus, as far as could be established electronically, all patients in this analysis had a period free of the drug of interest, commencement of the drug in question, and a period of equal duration subsequently. We calculated the number of admission days in the *M*_1_ and *M*_2_ periods.

As our interest was in the potential impact of treatments upon admissions, and the inclusion of large numbers of relatively well patients (having few admissions) would reduce power, we excluded all patients with zero admission days in *M*_1_ before comparing pre- and post-drug periods. Therefore, in this analysis we studied relatively unwell patients.

Overall admission rates decreased over time. If this effect were not itself due to treatments, then it would bias results in favor of treatments. We therefore corrected for overall admission rates. For each mirror period (*M*_1_ and *M*_2_), in addition to the per-subject admission rates *x*_1_ and *x*_2_, we calculated the mean admission rate for all patients, *μ*_1_ and *μ*_2_. Then, instead of analyzing (*x*_2_–*x*_1_), we analyzed and report [(*x*_2_–*μ*_2_)–(*x*_1_–*μ*_1_)].

### Regression analysis

All patients were analyzed simultaneously via a mixed-effects general linear model. We divided time into 6-month periods. The dependent variable was the number of inpatient days per patient per period (re-expressed as admission days per year). The predictors were: subject (a random factor), calendar period (to control for overall changes in admission rates; linear fixed effect), sex (factorial fixed effect), time since diagnosis (from the first recorded diagnosis of schizophrenia to the mid-point of the period in question, as an estimate of illness duration; linear fixed effect), age (though this correlates with time since diagnosis; linear fixed effect), and whether the patient was or was not documented as taking each drug in the immediately preceding period (factorial fixed effects; data from the very first period were therefore not modeled; total subject *n*=1,406). Subjects contributed only whole periods (not contributing to a period if it began before their first diagnosis of schizophrenia, or ended after their death). Analyses were fit using the R^18^ function lmerTest::lmer, and *P* values and 95% confidence intervals (CIs) were calculated using the Satterthwaite degrees-of-freedom approximation and Type III sums of squares (estimating the effect of each predictor over and above the effect of all others, particularly relevant where predictors are correlated).

Sulpiride, amisulpride, and mirtazapine are among drugs used for augmentation of treatment with clozapine,^19^ and co-prescription was common. We therefore added drug–drug interaction terms between clozapine and each of: sulpiride, amisulpride, mirtazapine, aripiprazole, and olanzapine (thereby including also interactions with other drugs associated with a reduction in admission days in the 2-year mirror analysis). As the clozapine×sulpiride and clozapine×mirtazapine interactions were far from significant (*P*>0.5), our final regression model included interactions between clozapine and each of amisulpride, aripiprazole, and olanzapine, plus all main effects. To exclude the possibility that the effects attributed to non-clozapine drugs were due to a confound with clozapine treatment or vice versa, we re-ran the regression for the subset of patients who had received clozapine at some point, or only those who had not received clozapine.

## Results

### Demographics

A total of 1,485 patients had coded diagnoses of schizophrenia (941 male, 544 female; sex difference, *χ* ^2^_1_=106, *P*=6.89×10^−25^). At the first electronically recorded diagnosis, the median age was 38 years (range 13–93 years, interquartile range 24 years, central 95% of patients in the range 18.0–77.3), likely reflecting a relatively high proportion of patients with chronic schizophrenia. The data did not permit the accurate differentiation of new-onset schizophrenia versus chronic schizophrenia recorded electronically for the first time. The median period of follow-up was 4.98 years (from the first to the last letter/admission within the time range, calculable for 1,310 patients). The sampling method was designed to be specific rather than sensitive, and excludes any patients with schizophrenia whose diagnosis was recorded without ICD-10 coding, or for whom ICD-10 diagnoses were recorded only in free-text documents. Overall admission rates for schizophrenia declined substantially over time (from a mean of 49.3 days/year/patient in 2005 to 10.2 in 2012).

### Drug usage and sex differences in prescribing

Table 1 shows drug use rates for antipsychotics and antidepressants. Clozapine, olanzapine, and depot risperidone were all prescribed proportionally more in males, with no other significant sex differences for antipsychotics. 30.1% of males were prescribed clozapine at some point, and 21.9% of females (*χ* ^2^ test, *P*=2.01×10^−6^). For olanzapine, these values were 42.1 and 34.4% (*P*=1.31×10^−9^); for depot risperidone, 8.0 and 4.2% (*P*=0.0071). Females were more likely to be prescribed amitriptyline (5.9% vs. 2.2%, uncorrected *P*=4.5×10^−4^), and citalopram (18.0% vs. 13.6%, *P*=0.0274), with no other significant sex differences for antidepressants. Supplementary Table 1 indicates frequency of consecutive or temporally close co-prescription between pairs of drugs, and of monotherapy, and shows all drugs included in the regression analysis.

### Precision and recall for clozapine

For clozapine, recall was 1.0. Patient precision was 0.96. Temporal precision was 0.85.

### Mirror analysis: relationship between drug use and admission

**Figure 2:**
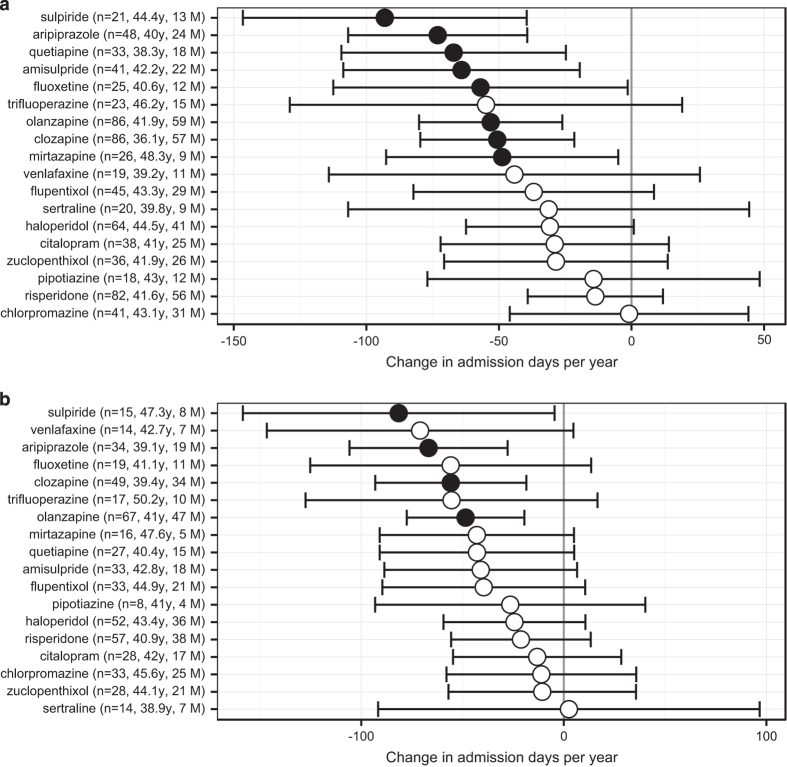
(**a**) One-year and (**b**) 2-year mirror-image analyses, showing the change in admission days per patient per year (admission days per year after drug, minus admission days per year before drug, corrected for overall admission rates; see Figure 1 and text). The number of subjects contributing to the measurement for each drug is shown in parentheses (*n*), with mean age in years (at first use of the drug in question, i.e., in the middle of the period considered) and the number of males (*M*). Points show means and error bars show 95% CIs; filled symbols indicate that the CI excludes zero.

Figure 2 shows the mirror-image analyses. Drugs associated with fewer subsequent admissions in the 1-year analysis were amisulpride, aripiprazole, clozapine, fluoxetine, mirtazapine, olanzapine, quetiapine, and sulpiride. Drugs that continued to be associated with fewer subsequent admissions in the more stringent 2-year analysis, despite fewer observations, were aripiprazole, clozapine, olanzapine, and sulpiride. In both analyses, sulpiride was associated with the largest reduction in subsequent admissions.

### Regression analysis

**Figure 3:**
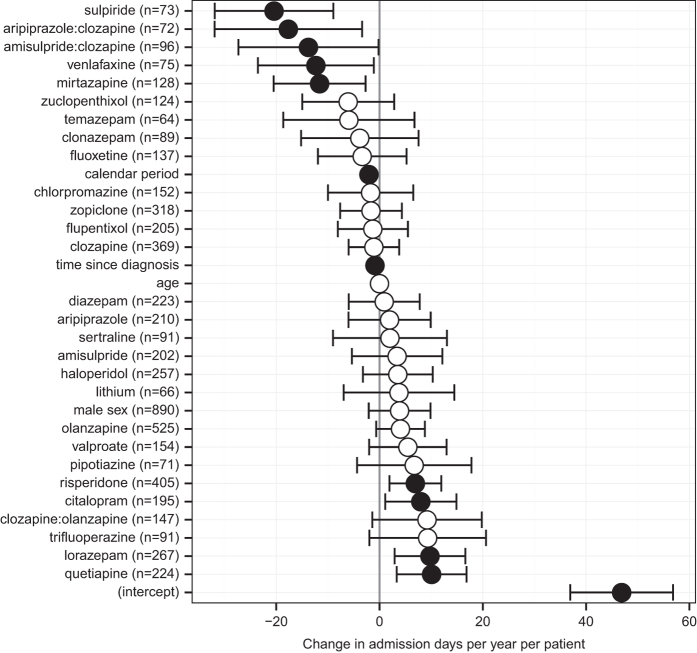
Association of admission rates during a given 6-month calendar period with drug use in the preceding calendar period (*n*=1,406). The results are expressed as a change in the number of admission days per patient per year (means±95% CI); filled symbols indicate that the CI excludes zero. Interaction terms are expressed with colon notation. The number of patients taking each drug in at least one time period (whether alone or with another drug) is shown in parentheses (*n*); for interactions, this is the number of patients who took both drugs during the same time period, for at least one period. The effect sizes were derived from a regression analysis taking account of all other antipsychotic and antidepressant drugs in the analysis (see text), plus 6-month calendar period number (to account for overall trends over time), age, time since the first recorded diagnosis of schizophrenia (in years), and sex.

In the regression analysis (Figure 3), sulpiride and mirtazapine continued to be associated with a reduction in admissions (estimated mean change −20.4 and −11.6 days/year, respectively). The changes were smaller than in the mirror analysis (expected since all patients were included and statistical control for other factors was better). Clozapine–aripiprazole and clozapine–amisulpride combinations were also associated with a significant decrease in admission rates, beyond the effect of either alone (interaction effects −17.7 and −13.8 days/year, respectively), as was venlafaxine (−12.3). As noted above, clozapine×sulpiride and clozapine×mirtazapine interactions were far from significant in preliminary analysis. Some antipsychotics were associated with an increase in admission rates, although this method of analysis may be biased against drug effectiveness (see Discussion). The overall mean admission rate was 26.8 days/year. Admission rates decreased with time, but, in addition, time since diagnosis was negatively associated with admissions.

Within patients who had received clozapine at some point (*n*=402), mirtazapine continued to be negatively associated with admission days (mean change −22.0 days/year, CI −42.2 to −1.8), while the effects of sulpiride or venlafaxine, while still negative, were not credibly different from zero in this subgroup (sulpiride −18.8, CI −37.7 to +0.2; venlafaxine, −12.4, CI −35.5 to +10.7). Within the group of patients who had not received clozapine (*n*=1,083), the effect of sulpiride remained strong (mean −19.9, CI −36.0 to −3.8), while the effects of mirtazapine and venlafaxine were not credibly different from zero (mirtazapine −4.7, CI −13.5 to +4.1, venlafaxine −8.7, CI −20.6 to +3.3).

Thus, the effect of mirtazapine was statistically independent of that of clozapine, though numerically stronger in clozapine users; clozapine and aripiprazole showed significant beneficial synergistic effects, as did clozapine and amisulpride; and sulpiride showed the largest beneficial effect overall, which was not solely due to its use as an augmentation strategy for clozapine.

## Discussion

In patients with schizophrenia, we found a significant decrease in admission days after initiation of sulpiride, aripiprazole, clozapine, and olanzapine, in mirror-image analyses covering 2 years before and after drug initiation. This analysis focused on patients with more severe disease, in that they had at least one hospital admission in the pre-drug period. Sulpiride, mirtazapine, venlafaxine, and clozapine–aripiprazole and clozapine–amisulpride combinations were associated with a decrease in subsequent time spent in hospital in a regression analysis across all 1,406 patients, controlling for the effects of other drugs. All analyses controlled for overall changes in admission rates unrelated to drug use. Some drugs in the regression analysis were associated with an increase in admissions; however, this method may be biased against drug efficacy (discussed below), so we do not rely on changes in this direction. In all analyses, the largest beneficial effects were associated with sulpiride, a drug used relatively infrequently in local practice.

### Methodological considerations

In the mirror-image design, we studied patients who were relatively unwell by considering only patients who had been admitted in the pre-drug period. Additional strengths of this analysis are that it examines a relatively long time period, represents a within-subjects comparison, and is an intention-to-treat analysis, since subjects who commenced and then stopped a given drug are not distinguished from those who started and continued—in this respect unlike^17^. A weakness of this method is the potential for influence by regression to the mean. If illness course fluctuates spontaneously and drugs are started when the illness is severe, natural variation will bias the results in favour of drug treatments. We attempted to reduce this by the use of the central exclusion gap but also by using both a temporally narrow mirror-image analysis (*M*=1 year) and a temporally broad analysis (*M*=2 years). Any effect of regression to the mean is less likely to be observed in the temporally broader analysis. An additional weakness of the mirror-image design is that it takes no account of other drugs given at the same time; we addressed this with a regression analysis.

The regression analysis accounted for the effects of all drugs considered simultaneously (examining the effects of each drug over and above all others), accounted for age and estimated illness duration (over and above calendar time), and examined drug–drug interactions. A weakness of this method is that *a priori* one might expect admissions to be associated with more frequent documentation in secondary care of a drug history. This would bias the analysis against effective drugs; therefore, we do not rely on effects in that direction. However, the analysis highlights drugs associated with the greatest decrease in admission days. Another weakness is that admissions may trigger the initiation of treatment (so causality might flow from admissions to drugs rather than the reverse).

### Comparison to other studies of efficacy and hospitalization in schizophrenia

Our results are broadly in line with previous evidence of antipsychotic efficacy as judged by changes in symptoms or hospitalization. A recent meta-analysis^20^ ranked clozapine, amisulpride, and olanzapine as the top three for improving symptoms of schizophrenia and for lack of all-cause discontinuation, a combination that might be expected to reduce hospitalization. Hospitalization benefits for clozapine and olanzapine have also been observed before. A double-blind randomized trial favored olanzapine over perphenazine, quetiapine, risperidone, or ziprasidone for rehospitalization,^2^ and a non-blind randomized trial favored clozapine over usual care.^3^ Non-randomized studies have found hospitalization advantages for clozapine, versus risperidone or SGAs collectively,^4^ FGAs,^5^ depot FGAs,^6^ or a pre-clozapine period.^7^ After a first hospitalization for schizophrenia/schizoaffective disorder, the lowest risk of rehospitalization was associated with the use of olanzapine, clozapine, and perphenazine depot.^8^ That study did not examine some drugs associated with fewer admission days in our analysis (sulpiride, aripiprazole, and mirtazapine) and perphenazine was not used sufficiently in our sample for analysis, but olanzapine and clozapine were associated with positive benefit in terms of hospitalization in both studies (at least, in our study, for patients having more frequent admissions within the mirror-image analysis).

Aripiprazole and amisulpride were associated with benefit when combined with clozapine. Aripiprazole monotherapy has not shown better efficacy than other antipsychotics on reducing readmission;^21^ however, its use for clozapine augmentation has shown benefit.^9,10^ Two small studies have shown some benefits for amisulpride as clozapine augmentation.^22,23^ Overall, 27.1% of patients in our study had been prescribed clozapine at some point, within the expected range.^24,25^

Mirtazapine was associated with fewer admission days, in analyses controlling for other drugs. This was statistically independent of clozapine. No studies have specifically examined the impact of mirtazapine or venlafaxine on admissions in schizophrenia; however, there is increasing (though heterogeneous) evidence that adding mirtazapine can improve negative/cognitive symptoms.^11^ Treatment of depression in schizophrenia might also be an important mode of action, though the effects of four other conventional antidepressants, flupentixol, or quetiapine upon admissions were not significant or not beneficial.

Perhaps the most striking result in our study was the consistent and large reduction in admissions associated with sulpiride. The CUtLASS 1 study^26^ found that SGAs were not superior to FGAs, favouring FGAs; of those FGAs, sulpiride was a common choice. A previous systematic review found little difference between sulpiride and other antipsychotics.^27^ However, a recent large epidemiological study found sulpiride to be associated with less discontinuation than haloperidol, risperidone, or olanzapine, and similarly less hospitalization, though the latter difference was not significant.^28^ There is also some evidence for the use of sulpiride as clozapine augmentation,^29^ though in our data the sulpiride effect did not interact with that of clozapine.

### Study strengths

This study has strengths as a naturalistic observational study across a health care provider, covering a wide range of patient ages and clinical teams, with natural variation in prescribing practice. The analytical methods examined the within-subject effects of all antipsychotics and antidepressants in common use, controlling statistically for the effects of the other drugs in use and for temporal factors.

### Study weaknesses

Amongst its several weaknesses, the study is observational, not interventional, so causal conclusions cannot be drawn. For example, a particular drug associated with better outcomes may itself be used preferentially by clinical teams that deliver better care, or be prescribed more for patients whose disease is more responsive to treatment, or some other such unmeasured confound. Conversely, a drug may be spuriously associated with worse outcomes because it is perceived to be clinically effective and is therefore prescribed for more severely ill patients at the start of a psychiatric admission.

Some drugs were recorded at median doses below the therapeutic minimum (Table 1), specifically chlorpromazine, pipotiazine, and clozapine. This represents a source of bias against these drugs’ effectiveness. In some cases the doses were systematically underestimated by the NLP-generated drug histories, notably for split-dose drugs such as clozapine.

In addition, the data are noisy and/or incomplete for several reasons, leading to a reduction in power and potential bias. There is likely to have been under recording of schizophrenia diagnoses, given the generally low rates of ICD-10 diagnostic coding. In this respect we preferred specificity over sensitivity, but it is possible that there was a systematic difference between patients with schizophrenia who did and did not have their diagnosis electronically coded. The temporal precision of NLP-generated drug histories was imperfect. Concomitant treatments (such as psychotherapy or electroconvulsive therapy) and concomitant physical illnesses were not measured and represent an additional source of noise and potential bias. As the drug data are based on NLP analysis of clinical documents representing snapshots of each patient when seen in secondary care, use of a given drug between snapshots was not measured. This weakness prohibits also accurate characterization of the duration of use of individual drugs, necessitating an intention-to-treat approach and likely lowering power (since drugs used only briefly are thus included), and did not permit an accurate characterization of illness course. Our data are of significantly lower quality than large data sets including electronic prescription and dispensing information;^30,31^ however, they may nonetheless provide novel suggestions.

The overall decline in admissions is likely to represent primarily service reorganizations leading to a shift in admission threshold, thought might also reflect a degree of artifact of loss to follow-up of patients moving to other geographical areas, or improvements in treatment.

## Conclusions

In an observational study of 1,485 patients with schizophrenia in a secondary mental health care setting, the initiation of sulpiride, mirtazapine, venlafaxine, or clozapine–aripiprazole and clozapine–amisulpride combinations was associated with a decrease in subsequent time spent in hospital, controlling for other drugs. In simple comparisons of admissions before and after each drug was started, sulpiride, aripiprazole, clozapine, and olanzapine were associated with a reduction in subsequent admissions, for patients admitted in the pre-drug period. The association of clozapine and olanzapine with a reduction in subsequent admissions has been observed before, as have some benefits for amisulpride and aripiprazole as clozapine augmentation, but the association of sulpiride, mirtazapine, and venlafaxine with these benefits is novel. Sulpiride was associated with the greatest reduction in inpatient days in all analyses, independently of clozapine use. These findings are not conclusive, do not imply causation, and should not be used to alter clinical practice but raise hypotheses requiring confirmation or refutation by randomized controlled trials.

## Figures and Tables

**Table 1:** Frequency of drug prescribing

*Drug*	*Proportion of all patients prescribed drug at some point during the study period (%)*	*Median daily dose (for drugs taken by ≥5% of patients)*	*Minimum effective daily dose* ^32^ *, where established*
*First-generation antipsychotics*			
Chlorpromazine	12.0	150 mg	200 mg^33^ a
Flupentixol	15.7	6 mg	?^34,35^
Fluphenazine	4.0	–	–
Haloperidol	20.1	10 mg	4 mg^32^
Levomepromazine	0.1	–	–
Pericyazine	0.1	–	–
Perphenazine	0.1	–	–
Pimozide	0.7	–	–
Pipotiazine	5.1	7.1 mg	21 mg^36^ a
Prochlorperazine	0.5	–	–
Promazine	0.4	–	–
Sulpiride	5.7	400 mg	400 mg^33^ a
Trifluoperazine	7.5	10 mg	10 mg^33^ a
Zuclopenthixol	8.8	37.9 mg	14 mg^37^ a
			
*Second-generation antipsychotics*			
Amisulpride	15.5	400 mg	400 mg^33^ a
Aripiprazole	16.8	15 mg	10 mg^32^
Asenapine	0.1	–	–
Clozapine	27.1	250 mg^b^	300 mg^32^
Olanzapine	39.3	12.5 mg	7.5 mg^32^
Paliperidone	2.4	–	–
Quetiapine	17.6	300 mg	150 mg^32^
Risperidone	31.9	3.6 mg	2 mg^32^
			
*Antidepressants used by ≥5% of patients*			
Citalopram	15.2	20 mg	20 mg^33^
Fluoxetine	10.8	20 mg	20 mg^33^
Mirtazapine	10.0	30 mg	30 mg^33^
Sertraline	7.3	100 mg	50 mg^33^
Venlafaxine	5.9	150 mg	75 mg^33^

Second-generation antipsychotics (SGAs) were in general used more than first-generation antipsychotics (FGAs), except for relatively frequent use of haloperidol (which is often prescribed ‘as required’ in this institution). The FGA benperidol and the SGAs iloperidone, lurasidone, sertindole, ziprasidone, and zotepine were not used. The percentages of patients prescribed a depot medication at some point were: *FGAs*: haloperidol 2.1%, flupentixol 12.8%, fluphenazine 3.2%, paliperidone 2.4%, pipotiazine 5.1%, zuclopenthixol 1.6%; *SGAs:* olanzapine 0.1%, and risperidone 6.6%.

a Estimate.

b Clozapine doses were in some cases electronically underestimated as this is a drug whose dose is split across the day; for example, ‘clozapine 200 mg in the morning, 200 mg at night’ was misinterpreted as 200 mg/day.
