# Supplementary material for: Association between antipsychotic/antidepressant drug treatments and hospital admissions in schizophrenia assessed using a mental health case register
Source: NPJ Schizophr. 2015 Oct 21;1:15035–. doi: 10.1038/npjschz.2015.35 (PMC4849458; doi:10.1038/npjschz.2015.35)
Supplement: Supplementary Information [file npjschz201535-s1.pdf]

Association between antipsychotic/antidepressant drug treatments and hospital admissions in schizophrenia assessed using a mental health case register

Cardinal RN, Savulich G, Mann LM, Fernández-Egea E

SUPPLEMENTARY MATERIALS

SUPPLEMENTARY METHODS

Recall and precision

Recall was defined as the probability of retrieving a record, given that it was relevant. For example, recall for patients taking clozapine would be P(electronically identified as taking clozapine | actually taking clozapine). Precision was defined as the probability of a record being relevant, given that it was retrieved. For example, precision for the same search would be P(actually taking clozapine | electronically identified as taking clozapine).

Recall and precision were assessed for clozapine, for which mandatory monitoring requirements meant that we could independently and unequivocally establish a subset of patients who had taken clozapine using electronic methods. *(1) Recall.* From the set of patients with schizophrenia, attendance at a clozapine monitoring clinic and thus clozapine usage was established by searching clinical documents for the phrase "Clozapine Review & Monitoring Sheet" (used in one of the clinic's template documents). From these patients, 100 were sampled at random; recall was assessed as the proportion of these patients judged by their automated drug history to have been on clozapine at some point. *(2) Precision.* Precision was assessed in two ways. First, patient precision: 100 patients were selected at random from those with schizophrenia whom the automated methods had identified as being on clozapine at some point. Their records were manually examined to verify whether they had indeed taken clozapine at some point. Second, temporal precision: 100 instances of automatically identified clozapine use were selected at random (across all patients), and the records manually inspected to see if clozapine had been taken at the time suggested by the automatic process.

SUPPLEMENTARY TABLE 1

Frequency of co-prescription for antipsychotics, antidepressants, mood stabilizers, and benzodiazepines/cyclopyrrolones used by ≥5% of patients. For each drug pair, the first figure indicates the number of patients who received both drugs during the same 6-month calendar period at least once, and the figure in parenthesis indicates the number of patients who received both drugs at some point in the period of study (not necessarily simultaneously). The "–" row/column indicates monotherapy, for which the first number gives instead the number of patients on the specified drug and none of the others listed in at least one calendar period, and the second number gives the number of patients who received the specified drug at some point and none of the other drugs at any point. AMS, amisulpride; ARP, aripiprazole; OLZ, olanzapine; QUE, quetiapine; RIS, risperidone; CLO, clozapine; CHL, chlorpromazine; FLP, flupentixol; HAL, haloperidol; PIP, pipotiazine; SUL, sulpiride; TRI, trifluoperazine; ZUC, zuclopenthixol; CIT, citalopram; FLX, fluoxetine; SRT, sertraline; MIR, mirtazapine; VEN, venlafaxine; TEM, temazepam; CLN, clonazepam; DIA, diazepam; LRZ, lorazepam; ZOP, zopiclone; VAL, valproate; LI, lithium.

	AMS	ARP	OLZ	QUE	RIS	CLO	CHL	FLP	HAL	PIP	SUL	TRI	ZUC
**AMS**	202:202	42:59	99:111	50:61	66:84	96:105	32:42	37:48	60:75	10:14	20:27	27:31	25:30
**ARP**	42:59	210:210	94:118	52:75	67:103	72:86	22:33	30:43	40:57	7:13	12:17	16:21	17:27
**OLZ**	99:111	94:118	525:525	100:123	180:215	147:169	79:94	100:122	132:157	33:42	36:42	55:64	64:76
**QUE**	50:61	52:75	100:123	224:224	82:110	69:82	31:43	44:55	61:73	11:18	12:16	25:33	25:32
**RIS**	66:84	67:103	180:215	82:110	405:405	105:132	47:64	67:83	102:126	22:32	21:25	35:43	38:57
**CLO**	96:105	72:86	147:169	69:82	105:132	369:369	57:67	56:70	101:116	18:22	44:48	36:40	45:54
**CHL**	32:42	22:33	79:94	31:43	47:64	57:67	152:152	42:44	56:64	13:19	17:18	30:32	30:31
**FLP**	37:48	30:43	100:122	44:55	67:83	56:70	42:44	205:205	64:75	12:16	19:21	37:39	36:41
**HAL**	60:75	40:57	132:157	61:73	102:126	101:116	56:64	64:75	257:257	18:28	25:26	32:37	52:63
**PIP**	10:14	7:13	33:42	11:18	22:32	18:22	13:19	12:16	18:28	71:71	5:6	4:7	8:13
**SUL**	20:27	12:17	36:42	12:16	21:25	44:48	17:18	19:21	25:26	5:6	73:73	14:14	13:16
**TRI**	27:31	16:21	55:64	25:33	35:43	36:40	30:32	37:39	32:37	4:7	14:14	91:91	23:24
**ZUC**	25:30	17:27	64:76	25:32	38:57	45:54	30:31	36:41	52:63	8:13	13:16	23:24	124:124
**CIT**	38:48	38:57	89:116	41:57	70:92	70:87	23:35	26:36	32:48	8:12	7:11	11:22	17:23
**FLX**	39:45	31:46	64:79	38:46	48:61	47:59	21:28	22:31	33:40	6:8	10:11	17:21	14:18
**SRT**	23:27	14:27	50:62	24:31	34:42	31:37	10:12	19:24	28:31	2:6	7:9	13:14	13:14
**MIR**	25:29	29:37	58:73	31:40	48:64	34:40	12:19	27:30	30:38	2:3	6:9	9:16	16:18
**VEN**	13:20	19:31	38:54	18:22	27:40	24:32	7:12	9:14	14:21	1:2	3:8	6:13	9:15
**TEM**	17:22	8:16	28:38	23:29	18:25	23:28	17:20	13:19	19:24	5:8	6:7	8:10	7:10
**CLN**	10:18	24:27	37:53	18:29	35:49	31:39	11:15	12:25	35:46	4:8	3:4	6:14	15:21
**DIA**	48:61	46:69	123:150	56:71	93:115	81:93	44:53	49:57	73:90	17:23	12:18	27:32	32:37
**LRZ**	55:74	52:74	134:169	56:75	100:127	84:114	51:63	50:71	123:133	13:23	21:25	27:38	34:52
**ZOP**	77:88	65:95	160:187	65:90	119:146	111:132	47:58	52:73	87:110	14:24	14:19	23:31	31:43
**VAL**	26:33	30:41	76:85	41:49	57:71	59:70	20:27	27:37	51:60	12:19	12:13	12:15	18:23
**LI**	7:13	7:14	31:38	13:17	11:19	24:26	20:25	14:19	19:26	5:7	8:9	7:12	8:12
**-**	22:6	43:4	147:31	31:5	111:26	142:19	10:2	46:6	22:2	17:0	3:1	7:1	19:1

	CIT	FLX	SRT	MIR	VEN	TEM	CLN	DIA	LRZ	ZOP	VAL	LI	-
**AMS**	38:48	39:45	23:27	25:29	13:20	17:22	10:18	48:61	55:74	77:88	26:33	7:13	22:6
**ARP**	38:57	31:46	14:27	29:37	19:31	8:16	24:27	46:69	52:74	65:95	30:41	7:14	43:4
**OLZ**	89:116	64:79	50:62	58:73	38:54	28:38	37:53	123:150	134:169	160:187	76:85	31:38	147:31
**QUE**	41:57	38:46	24:31	31:40	18:22	23:29	18:29	56:71	56:75	65:90	41:49	13:17	31:5
**RIS**	70:92	48:61	34:42	48:64	27:40	18:25	35:49	93:115	100:127	119:146	57:71	11:19	111:26
**CLO**	70:87	47:59	31:37	34:40	24:32	23:28	31:39	81:93	84:114	111:132	59:70	24:26	142:19
**CHL**	23:35	21:28	10:12	12:19	7:12	17:20	11:15	44:53	51:63	47:58	20:27	20:25	10:2
**FLP**	26:36	22:31	19:24	27:30	9:14	13:19	12:25	49:57	50:71	52:73	27:37	14:19	46:6
**HAL**	32:48	33:40	28:31	30:38	14:21	19:24	35:46	73:90	123:133	87:110	51:60	19:26	22:2
**PIP**	8:12	6:8	2:6	2:3	1:2	5:8	4:8	17:23	13:23	14:24	12:19	5:7	17:0
**SUL**	7:11	10:11	7:9	6:9	3:8	6:7	3:4	12:18	21:25	14:19	12:13	8:9	3:1
**TRI**	11:22	17:21	13:14	9:16	6:13	8:10	6:14	27:32	27:38	23:31	12:15	7:12	7:1
**ZUC**	17:23	14:18	13:14	16:18	9:15	7:10	15:21	32:37	34:52	31:43	18:23	8:12	19:1
**CIT**	195:195	27:39	19:29	24:35	16:28	11:16	19:20	47:61	28:52	69:89	27:34	9:15	20:2
**FLX**	27:39	137:137	17:24	27:35	10:18	12:17	8:15	34:44	34:48	42:54	21:29	11:13	8:2
**SRT**	19:29	17:24	91:91	17:24	10:17	4:7	7:15	30:35	21:32	33:42	12:16	5:8	2:0
**MIR**	24:35	27:35	17:24	128:128	16:22	6:7	11:17	32:41	31:45	46:57	16:23	13:14	10:1
**VEN**	16:28	10:18	10:17	16:22	75:75	8:13	5:9	26:35	23:37	28:37	11:17	7:9	7:0
**TEM**	11:16	12:17	4:7	6:7	8:13	64:64	9:12	25:31	17:24	19:30	12:17	3:3	1:1
**CLN**	19:20	8:15	7:15	11:17	5:9	9:12	89:89	18:33	34:45	27:41	32:35	6:8	2:1
**DIA**	47:61	34:44	30:35	32:41	26:35	25:31	18:33	223:223	63:98	95:117	44:56	14:22	17:1
**LRZ**	28:52	34:48	21:32	31:45	23:37	17:24	34:45	63:98	267:267	108:131	49:65	17:24	9:1
**ZOP**	69:89	42:54	33:42	46:57	28:37	19:30	27:41	95:117	108:131	318:318	61:69	22:27	10:1
**VAL**	27:34	21:29	12:16	16:23	11:17	12:17	32:35	44:56	49:65	61:69	154:154	20:23	16:0
**LI**	9:15	11:13	5:8	13:14	7:9	3:3	6:8	14:22	17:24	22:27	20:23	66:66	4:1
**-**	20:2	8:2	2:0	10:1	7:0	1:1	2:1	17:1	9:1	10:1	16:0	4:1	-:-
